# Perinatal dengue and Zika virus cross-sectional seroprevalence and maternal-fetal outcomes among El Salvadoran women presenting for labor-and-delivery

**DOI:** 10.1186/s40748-024-00177-5

**Published:** 2024-04-02

**Authors:** Mary K. Lynn, Marvin Stanley Rodriguez Aquino, Pamela Michelle Cornejo Rivas, Xiomara Miranda, David F. Torres-Romero, Hanson Cowan, Madeleine M. Meyer, Willber David Castro Godoy, Mufaro Kanyangarara, Stella C.W. Self, Berry A. Campbell, Melissa S. Nolan

**Affiliations:** 1 Department of Epidemiology and Biostatistics, Arnold School of Public Health, University of South Carolina, 915 Greene Street #327, 29201 Columbia, SC USA; 2 Health Research and Development Center (CENSALUD), University of El Salvador, San Salvador, El Salvador; 3 Hospital Nacional “Dr Jorge Mazzini Villacorta”, Ministerio de Salud, Sonsonate, El Salvador; 4 Department of Chemistry and Pharmacy, University of El Salvador, Sonsonate, El Salvador; 5 Department of Obstetrics and Gynecology, Prisma Health, Columbia, SC USA

**Keywords:** Zika virus, Dengue virus, El Salvador, Parturition, Maternal-fetal medicine, Health outcomes, Health disparities, Flavivirus

## Abstract

**Background:**

Despite maternal flavivirus infections’ linkage to severe maternal and fetal outcomes, surveillance during pregnancy remains limited globally. Further complicating maternal screening for these potentially teratogenic pathogens is the overwhelming subclinical nature of acute infection. This study aimed to understand perinatal and neonatal risk for poor health outcomes associated with flaviviral infection during pregnancy in El Salvador.

**Methods:**

Banked serologic samples and clinical results obtained from women presenting for labor and delivery at a national referent hospital in western El Salvador March to September 2022 were used for this study. 198 samples were screened for dengue and Zika virus IgM, and statistical analyses analyzed demographic and clinical outcome associations with IgM positivity.

**Results:**

This serosurvey revealed a high rate of maternal flavivirus infection—24.2% of women presenting for labor and delivery were dengue or Zika virus IgM positive, suggesting potential infection within pregnancy. Specifically, 20.2% were Zika virus IgM positive, 1.5% were dengue virus IgM positive, and 2.5% were both dengue and Zika virus IgM positive. Women whose home had received mosquito abatement assistance within the last year by the ministry of health were 70% less likely to test IgM positive (aOR = 0.30, 95%CI: 0.10, 0.83). Further, statistical geospatial clustering revealed transmission foci in six primary municipalities. Pregnancy complications and poor birth outcomes were noted among the dengue and/or Zika virus maternal infection group, although these outcomes were not statistically different than the seronegative group. None of the resulting neonates born during this study were diagnosed with congenital Zika syndrome.

**Conclusions:**

The high rate of Zika virus detected among pregnant women and the lack of Zika-specific neonatal outcomes monitoring during a non-outbreak year highlights the need for continued surveillance in Central America and among immigrant mothers presenting for childbirth from these countries. As changing climatic conditions continue to expand the range of the disease vector, asymptomatic screening programs could be vital to early identification of outbreaks and clinical management of cases.

**Supplementary Information:**

The online version contains supplementary material available at 10.1186/s40748-024-00177-5.

## Background

The Latin America Zika virus (ZIKV) outbreak of 2015–2016 provided evidence for the causal relationships between congenital birth defects and flavivirus vertical transmission for the first time [1, 2]. Though cases of congenital Zika syndrome have since lessened, the disease is now endemic in Latin America and major questions remain regarding pathogenesis and vertical transmission risk [3]. The current prevalence of maternal and congenital infections are difficult to assess due to high rates of asymptomatic presentation and diagnostic cross-reactivity, particularly in resource limited settings [3–5]. Along with ZIKV, dengue virus (DENV), also in the family *Flaviviradae*, has a similar viral structure but unique pathogenesis [6]. Four distinct serotypes of DENV co-circulate in many regions, with increased risk of severe dengue upon secondary infection with a new serotype [7]. Both pathogens are primarily transmitted through the bite of the highly urbanized, female *Aedes aegypti* mosquito, are 75–80% asymptomatic, and endemic in Central America [1, 8, 9]—ZIKV is additionally transmitted sexually [5]. ZIKV may be transmitted vertically, causing pregnancy complications and poor maternal and neonatal health outcomes, that in severe cases, lead to life-long impairment of the corresponding neonate [6, 7, 10]. Though likely rare, DENV is also vertically transmitted [11, 12]. Vertical transmission evidence is primarily limited to case reports, and neonatal health outcomes are still being clarified [13]. Global travel and the potential for future arboviral outbreaks in the context of climate change begets the need for further clarification of the extent and potential disease impacts of ZIKV and DENV during pregnancy and congenital infections [5].

Congenital Zika syndrome is characterized by microcephaly and severe brain and central nervous system anomalies causing lifetime morbidity [6]. Though microcephaly is the traditional hallmark of infection, it is unclear how often this outcome occurs among congenital cases [14]. Further clinical manifestations are vast, including various cranial and brain anomalies, intracerebral and intrahepatic calcifications, hypertonicity, seizures, arthrogryposis, vision and hearing impairment, ventriculomegaly, diaphragm paralysis, and small for gestational age neonates [5, 6, 15]. Vertical transmission of ZIKV occurs in 1–13% of maternal infections, and severe sequelae are associated with early infections primarily in the first and second trimester [1, 15]. Few studies have investigated vertically transmitted dengue; however, DENV infection in pregnancy has been associated with increased risk for severe dengue outcomes including hypovolemic shock and hemorrhage, as well as three times the risk of maternal mortality [2, 7, 16]. Intrauterine growth restriction, premature placental detachment, preterm birth, miscarriage, and stillbirth have been associated with maternal infection [2, 10, 17]. Vertical transmission is thought to occur with infections in later pregnancy and yield varied manifestations in the neonate, ranging from subclinical infections to dengue hemorrhagic fever and dengue shock syndrome [18, 19]. Prior DENV infections have shown protective effects against ZIKV congenital infection in human studies; however, animal models have demonstrated an enhanced risk of congenital transmission in this scenario [20]. Further, increased risk of severe dengue has been associated with prior ZIKV infection [15, 20]—a very real epidemiologic concerns in Latin America.

In El Salvador, high population density coupled with high rates of land use change and deforestation create the ideal ecological niche for the persistence of *Aedes* mosquito breeding habitats and flavivirus replication [4, 21]. DENV and ZIKV viruses circulate annually in this country, with an annual variance in flavivirus dominance [22, 23], and cases are monitored via syndromic reporting to the Ministry of Health [24, 25]. However, due to the commonality of subclinical infection, coupled with limited national healthcare resources for arboviral laboratory diagnostics and poor access to care in rural regions, the true annual arbovirus burden and veritable agent are difficult to assess, and likely underreported or misreported. In 2022, the El Salvador Ministry of Health reported 172 suspected ZIKV cases and 16,542 suspected DENV cases [26]—the majority of these cases being syndromically diagnosed without formal laboratory confirmation.

Given the knowledge gap surrounding the infection burden and pathogenic impact of these two public health important flaviviruses in El Salvador, particularly among pregnant women, this study aimed to examine the Zika and Dengue IgM seroprevalence in a parturition cohort at a large reference hospital in western El Salvador. Further, we evaluated the impact of maternal flavivirus IgM presence on pregnancy and birth outcomes among women and their neonates at the time of labor and delivery.

## Methods

This project utilized a set of banked clinical samples to measure the acute antibody prevalence of ZIKV and DENV among women presenting for childbirth at the largest public referent hospital in the western department of Sonsonate, El Salvador. Detailed methods of the original cross-sectional study are described in Lynn et al. 2023 [26]. In brief, patients presenting for labor and delivery were recruited from Hospital Nacional General “Dr. Jorge Mazzini Villacorta” (Hospital Mazzini) as part of a congenital Chagas disease study. From March to September 2022, the months when most mosquito-borne diseases occur (the rainy season is May to October), 198 patients aged ≥ 15 years were recruited for the original study. All participants provided written informed consent, and those < 18 years provided written informed consent with parent/guardian written informed assent. Permission to use banked samples for further infectious disease testing was obtained in the original informed consent documents, and through the National Ethics Committee of El Salvador. After informed consent and/or assent, participants donated a small blood sample for pathogen serologic and molecular testing and a urine sample to assess preeclampsia indicators. Participants completed a lifetime risk factor questionnaire for vector-borne disease infection. Perinatal and neonatal health outcomes were also collected to assess complications during pregnancy and at parturition. Maternal banked sera for these 198 participants were available for flavivirus testing and perinatal health records were available for epidemiologic-clinical analysis.

### Ethics statement

The original study and the use of its banked samples for arbovirus testing were approved by the National Ethics Committee of El Salvador (project IRB Nº 0005660). Participants further provided written informed consent for blood samples to be used for expanded infectious disease testing, and samples were temporarily stored at the University of El Salvador (San Salvador, El Salvador), then biobanked at the University of South Carolina’s Laboratory of Vector-Borne and Zoonotic diseases (Columbia, SC, USA).

### Serologic and molecular methods

To determine the presence of IgM antibodies to ZIKV and DENV, serum samples were processed using enzyme-linked immunosorbent assay via the anti-ZIKV virus IgM ELISA and anti-DENV virus IgM ELISA kits (Calbiotech, El Cajon, CA, USA). Cross-reactivity rate was not provided in either kit’s instruction leaflet. However, no cross-reactivity was detected with in-house tests of positive controls provided with the kits.

### Statistical analysis

For the purposes of this analysis, we defined one positive category by general flavivirus IgM positivity: DENV and/or ZIKV IgM+. Fisher’s exact test was employed to determine univariate relationships between flavivirus infection (DENV and/or ZIKV IgM+) and (1) infection risk factors and (2) maternal and neonatal health outcomes. Multivariable logistic regression was used to determine statistically significant associations and odds ratios for the relationship between DENV and/or ZIKV IgM+, and infection risk factors or health outcomes. All infection risk factors, perinatal, and neonatal health outcomes were obtained from the original study survey. Regression models for infection risk included variables from the univariate analysis listed in Table 1, as well as reported histories of being sick, remarkable insect bite, rash, and fever in the last year. Maternal and neonatal health outcome models included those listed in Table 2. Models used complete case analysis when any of the included variables had > 5% missingness; therefore, the infection risk factor model contained 137 observations while all 198 observations were included in the health outcomes model. All statistical analyses were performed using RStudio version 4.1.1 (RStudio, PBC, Boston, MA).

Cluster and Outlier Analysis (via Anselin Moran’s I) was used to identify geospatial hot spots, cold spots, and outliers for disease categories. To account for differences in participant recruitment across municipalities, the proportion of positive cases out of the total number of individuals tested per municipality was used for this analysis. Spatial statistical analysis was performed using ArcGIS Pro (Version 2.4, Esri Inc.).

## Results

**Fig. 1:**
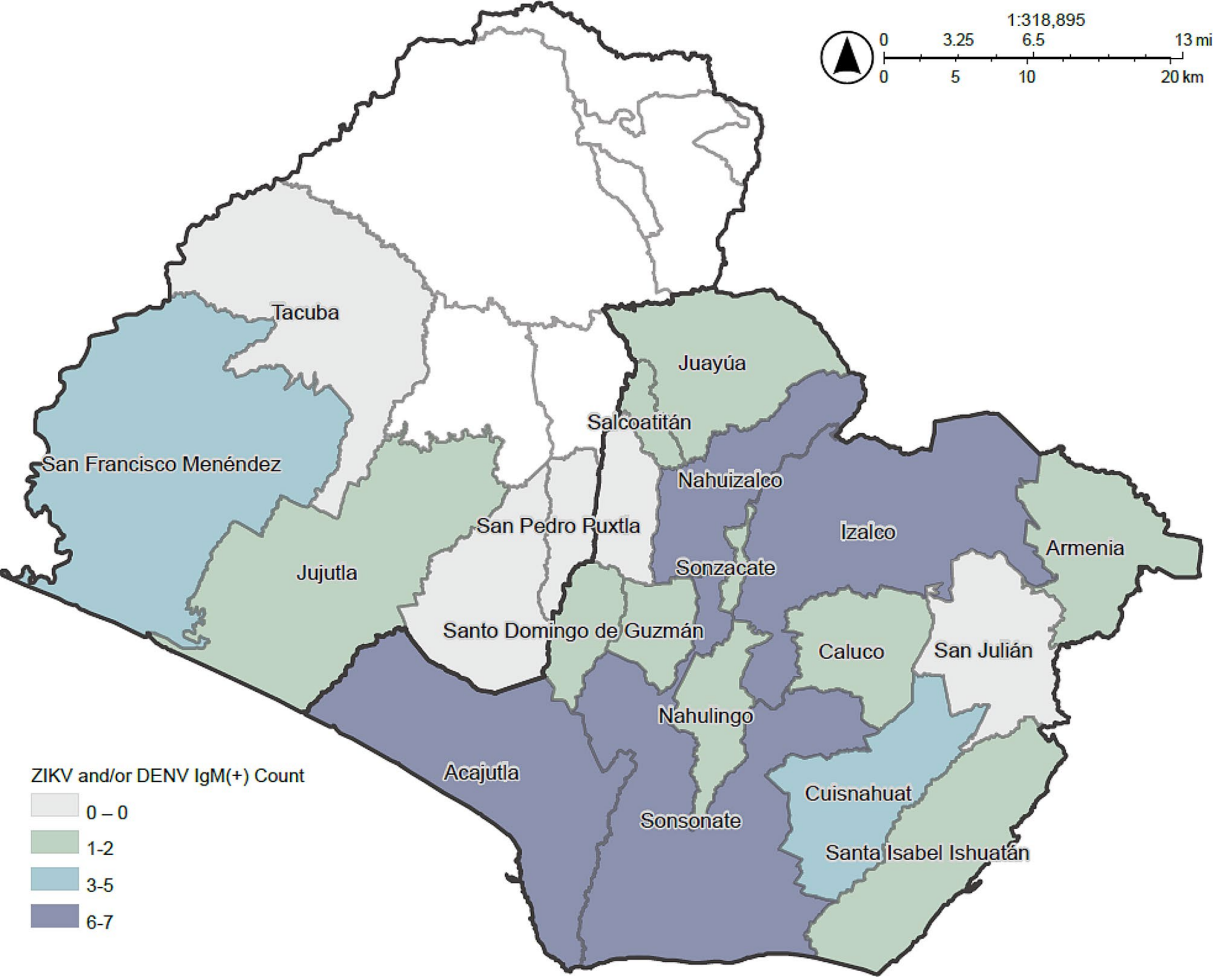
Case counts of perinatal DENV and/or ZIKV IgM + by municipality, Ahuachapan (left) and Sonsonate (right) departments. Municipalities with the highest DENV and/or ZIKV IgM + were found in Acajutla, Izalco, and Sonsonate (*N* = 7). *Municipalities colored in white indicate no participants from the original study stemmed from these areas; therefore no data was available

**Fig. 2:**
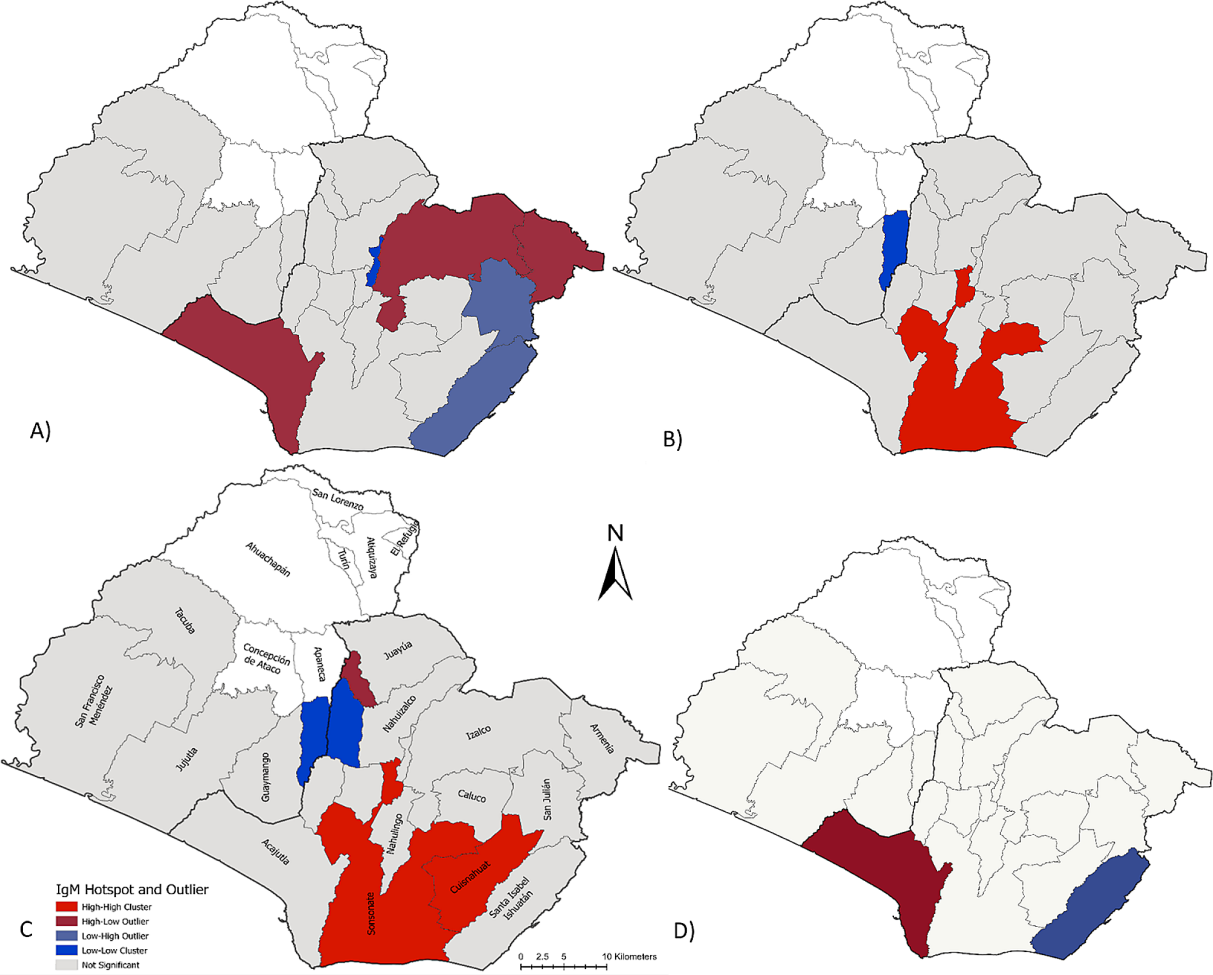
Cluster and Outlier Analysis of arboviral IgM positivity based on proportional positivity by recruitment municipality. **A)** DENV IgM+, **B)** ZIKV IgM+, **C)** DENV and/or ZIKV IgM+, **D)** DENV and ZIKV IgM+. High-High clusters represent areas with spatially significantly higher proportion of cases per municipality compared to surrounding municipalities. Low-Low clusters represent areas with spatially significantly lower proportion of cases per municipality compared to surrounding municipalities. High-Low Outliers indicate municipalities with comparatively higher flavivirus positive prevalence from our participant population surrounded by municipalities of lower prevalence. Low-High Outliers indicate areas of lower flavivirus positive prevalence from our study population surrounded by municipalities with comparatively higher prevalence

We found 24.2% (*N* = 48) of perinatal serum samples were IgM positive for at least one flavivirus infection, as shown in Fig. 1. Approximately 2.5% (*N* = 3) of samples had evidence of IgM antibody response to DENV alone and 20.2% (*N* = 40) had evidence of IgM antibody response to ZIKV alone. Further, 2.5% (*N* = 5) of serum samples showed IgM seropositivity to both pathogens concurrently. Among those positive for ZIKV and/or DENV IgM, the majority of cases occurred in Acajutla, Izalco, and Sonsonate (*N* = 7), shown in Fig. 1. The majority of ZIKV IgM positive only cases came from Nahuizalco and Izalco (*N* = 6), while DENV IgM positive only case counts were dispersed in Armenia, Izalco, and Sonsonate (*N* = 1) of Sonsonate department Detailed case counts and proportional positivity by department and municipality are available in Additional File 1. Spatial statistics revealed distinct clusters and outliers that followed a different pattern than the simple case counts for each pathogen, as noted in Fig. 2, with specific geospatial clusters highlighting potential transmission foci.

Demographics of the participant population and clinical histories are described in Table 1. In general, this cross-sectional perinatal group was healthy, with a small number reporting history of pregnancy complications (7.6%, *N* = 15) or their current pregnancy being classified as high-risk (16.7%, *N* = 33). Education levels ranged among the group with the majority of women having less than 7th grade education (43.4%, *N* = 86). Most lived in substandard conditions, as indicated by homes with barren earth floors (26.8%, *N* = 53), no electricity (7.5%, *N* = 15), and/or no potable water source (2.5%, *N* = 5). Over one quarter of women lived in high-risk areas for vector-borne disease, as evidenced by reported Ministry of Health (MOH) fumigation intervention at the home within the last year (26.8%, *N* = 53). Similarly, > 25% of the participant population had an MOH visit to the home in the last year to provide mosquito abatement assistance. Most perinatal women did not report frequently seeing mosquitos inside the home; however, the majority slept beneath insecticide-treated bed nets (ITNs) (62.1%, *N* = 123). Women reported using ITNs for various periods of time, ranging from 6 months to 15 years, and some reported only using ITNs during specific seasons throughout the year.

**Table 1:** Participant population demographics and results of fisher’s exact test based on DENV and/or ZIKV seropositivity

Descriptor	DENV and/or ZIKV IgM+ (*N* = 48) %(N)	DENV and ZIKV IgM- (*N* = 150) %(N)	Total population (*N* = 198) %(N)	*P-value Fisher’s
Department				0.506
Ahuachapan	12.5% (6)	17.3% (26)	16.2% (32)	
Sonsonate	87.5% (42)	82.7% (124)	83.8% (166)	
Age Group				0.033
< 18 years	10.4% (5)	6.0% (9)	7.1% (14)	
18–25 years	47.9% (23)	42.7% (64)	43.9% (87)	
26–30 years	29.2% (14)	19.3% (29)	21.7% (43)	
31–45 years	12.5% (6)	32.0% (48)	27.3% (54)	
History of prior miscarriage				1.0
No	89.6% (43)	89.3% (134)	89.4% (177)	
Yes	10.4% (5)	10.7% (16)	10.6% (21)	
High Risk Pregnancy (current)				1.0
No	83.3% (40)	83.3% (125)	83.3% (165)	
Yes	16.7% (8)	16.7% (25)	16.7% (33)	
Prior pregnancy complications (any)				0.366
No	89.6% (43)	93.3% (140)	92.4% (183)	
Yes	10.4% (5)	6.7% (10)	7.6% (15)	
Mother’s education level				1.0
< High school	68.8% (33)	69.3% (104)	69.2% (137)	
High School or greater	31.3% (15)	30.7% (46)	30.8% (61)	
Number of children*				1.0
none	37.5% (18)	36.9% (55)	37.1% (73)	
1–3	60.4% (29)	59.1% (88)	59.4% (117)	
4+	2.1% (1)	4.0% (6)	3.6% (7)	
Significant substandard housing*				0.470
No	75.0% (36)	68.5% (102)	70.1% (138)	
Yes	25.0% (12)	31.5% (47)	29.9% (59)	
Total number in household*				0.667
< 3	18.8% (9)	20.1% (30)	19.8% (39)	
4–6	72.9% (35)	66.4% (99)	68.0% (134)	
7+	8.3% (4)	13.4% (20)	12.2% (24)	
Reported mosquitoes typically in the home*				0.785
No	89.6% (43)	90.5% (134)	90.3% (177)	
Yes	10.4% (5)	9.5% (14)	9.7% (19)	
Insecticide-treated bed net (ITN) use*				0.499
No	41.7% (20)	36.2% (54)	37.6% (74)	
Yes	58.3% (28)	63.8% (95)	62.4% (123)	
# ITNs in the household*				0.929
none	9.7% (3)	10.4% (11)	10.2% (14)	
1–3	83.9% (26)	80.2% (85)	81.0% (111)	
4+	6.5% (2)	9.4% (10)	8.8% (12)	
Reported MOH visit in last year for fumigation*				0.041
No	47.1% (16)	67.9% (74)	62.9% (90)	
Yes	52.9% (18)	32.1% (35)	37.1% (53)	
Reported MOH visit in last year for mosquito abatement*				0.298
No	71.9% (23)	59.8% (64)	62.6% (87)	
Yes	28.1% (9)	40.2% (43)	37.4% (52)	

*some survey questions were left blank by participants therefore not all totals = 198; %(N) DENV and/or ZIKV IgM + refer to 48 positive for either or both DENV IgM and ZIKV IgM

Perinatal women in this study were highly likely to have cesarean section births (55.1%, *N* = 109), and pregnancy complications were mostly mild, shown in Table 2. Among neonates born to enrolled mothers, approximately 6% were born premature, nearly 8% were low birthweight (< 2,500 g) and 20% were sent to the neonatal intensive care unit (NICU) for varying reasons including low birthweight, low APGAR scores, MAS and ARDS. None of the neonates born to enrolled women in this study were reported with microcephaly or remarkable cranial abnormalities at birth. Three women and their neonates were transferred to higher capacity clinical care in the capital city due to complications including fetal deterioration and prenatal asphyxia.

**Table 2:** Participant and neonatal health outcomes with fisher’s exact test results for association

	% (N) DENV and/or ZIKV IgM+ (*N* = 48)	% (N) DENV and ZIKV IgM- (*N* = 150)	% (N) study population (*N* = 198)	*P-value Fisher’s
Pregnancy Outcomes				
Any complications	16.7% (8)	24.0% (36)	22.2% (44)	0.325
Gestational Diabetes	0% (0)	2.7% (4)	2.0% (4)	0.574
Threat of miscarriage	12.5% (6)	8.7% (13)	9.6% (19)	0.411
Placenta previa or acreta	0% (0)	2.7% (4)	2.0% (4)	0.574
Breech birth	4.2% (2)	4.0% (6)	4.0% (8)	1.0
Urinary tract infection (UTI) during pregnancy	2.1% (1)	2.7% (4)	2.5% (5)	1.0
Labor and Delivery Outcomes				
Cesarean section (c-section)	43.8% (21)	58.7% (88)	55.1% (109)	0.125
Any Complication	6.3% (3)	13.3% (20)	11.6% (23)	0.296
Premature rupture of membranes (PROM)	4.2% (2)	6.0% (9)	5.6% (11)	1.0
Neonatal Birth Outcomes				
Any symptom	6.25% (3)	11.3% (17)	10.1% (20)	0.416
Low birthweight	8.3% (4)	7.3% (11)	7.6% (15)	0.757
Apgar 1 min < 9	8.3% (4)	6.7% (10)	7.1% (14)	0.746
Apgar 5 min < 9	0% (0)	2.0% (3)	1.5% (3)	1.0
Admitted to neonatal intensive care (NICU)	12.5% (6)	22.7% (34)	20.2% (40)	0.151
Premature birth	10.4% (5)	4.0% (6)	5.6% (11)	0.135
Meconium aspiration syndrome (MAS)	0% (0)	3.3% (5)	2.5% (5)	0.593
Acute respiratory distress syndrome (ARDS)	4.2% (2)	6.0% (9)	5.6% (11)	1.0
Respiratory assistance (hood)	4.2% (2)	6.0% (9)	5.6% (11)	1.0

*Apart from those listed in the table: Any pregnancy complications included vaginal condylomatosis, loss of amniotic fluid, pyelonephritis, fetal deterioration, and other rare outcomes among this population. *Any labor and delivery complications included mother with vaginal infection at the time of birth and neonatal injury at birth.*Any symptom for neonatal birth outcomes included neonatal asphyxia, fetal macrosomia, and others that were rare outcomes in this study

Among DENV and/or ZIKV IgM + perinatal women, nearly 17% had pregnancy complications. Approximately 10% of neonates born to DENV and/or ZIKV IgM + mothers were premature, 8% had low birthweight, and nearly 13% were admitted to the NICU. Univariate analysis revealed significant relationships between DENV and/or ZIKV IgM + women and having MOH fumigation intervention at their household within the last year (*p* = 0.04) and maternal age group (*p* = 0.033). Multivariable logistic regression revealed that DENV and/or ZIKV IgM + women were significantly more likely to have MOH fumigation within their household within the last year (aOR = 4.73, 95%CI: 1.78, 13.43) and MOH mosquito abatement assistance in the last year (aOR = 0.30, 95%CI: 0.10, 0.83). No significant associations were identified between DENV and/or ZIKV IgM + and maternal or neonatal health outcomes.

## Discussion

This study identified evidence of elevated arboviral infection in a cohort of parturition mothers at a national referent hospital in western El Salvador. Approximately 24.2% were DENV and/or ZIKV IgM + at the time of childbirth, suggesting arboviral infection during their pregnancy. The majority of seropositive flavivirus cases came from Sonsonate department, where spatially significant clusters were identified in Sonsonate and Cuisnahuat municipalities, Fig. 2C. This result is consistent with 2022 MOH reports of elevated risk for infection in Sonsonate municipality [26]. We also identified a spatial outlier, a municipality with a significantly higher proportion of cases than neighboring municipalities, in Salcoatítan.

Results of this study further revealed that those participants who had MOH fumigation within the home in the last year had approximately 5 times the risk of seropositivity, compared to those who did not report MOH fumigation. MOH fumigation services are targeted neighborhood-level efforts to eliminate *Aedes spp.* mosquitoes along with other infectious disease vectors, following suspected or confirmed human vector-borne infection. In contrast, participants who were visited by the MOH for mosquito abatement assistance were 70% less likely to be flavivirus IgM seropositive. As hypothesized, spatial proximity to known cases of vector-borne infection in areas already vulnerable to these diseases was a risk factor for IgM + in this study. Mosquito abatement methods typically include insecticide spraying, larvicide donations for commonly used water storage containers, and environmental control (e.g. removal of mosquito breeding habitats). These results indicate favorable reach of the MOH mosquito abatement campaigns, and that enhanced abatement and fumigation efforts should be targeted in neighborhoods with historically high arboviral case burdens.

Pregnancy complications and adverse neonatal birth outcomes including premature birth, low birthweight, and NICU admission, occurred frequently among flavivirus seropositive mothers. However, no statistically significant relationships were found among IgM positivity and adverse health outcomes. Adverse neonatal outcomes were also frequent among seronegative mothers in this study, making clarification of flavivirus IgM + cases and health outcomes challenging.

No statistically significant associations were found between assessed poverty indicators: water source in the home, housing materials, ITN use, or substandard housing. However, this particular region is highly economically vulnerable with homogenous substandard living conditions, and a high multidimensional poverty index above national averages [27]. Ahuachapan and Sonsonate rank among the highest in country for inadequate housing materials, food insecurity, and crowding [27]. Over 25% of houses in this region lack access to potable water and more than 50% lack access to sanitation services [27]. Therefore, precise socioeconomic and standard of living comparisons were difficult to compare between groups due to the highly underserved nature of the entire study population. Given the overall high rate of multidimensional poverty in this region and the substandard living conditions that yield supportive mosquito breeding habitats, the risk of arboviral infection exists in this particularly vulnerable national region.

Despite ZIKV case reports significantly lower in national surveillance reporting compared to DENV nationally, our study found a high rate of ZIKV IgM + in perinatal women [23]. A 2019 study modelling ZIKV transmission risk in Latin America using a maximum entropy approach identified El Salvador to be among a few countries in the region at particularly high risk for transmission [21]. In the same year of our study, 2022, the Ministry of Health reported 172 ZIKV cases with 6 cases reported among pregnant women in the country: one of these six cases occurred in Sonsonate. As nearly 80% of ZIKV infections present asymptomatically, it is likely the incidence of infection is higher than MOH reports based on clinical syndrome. Further, 62 cases of ZIKV were identified among infants < 1 year of age [24, 26], shown in Additional File 2. This highlights the potential that a proportion of these cases may have resulted from congenital transmission.

The challenges with serologic cross-reactivity between these two infections is also well known [28]. Therefore, it is possible that some ZIKV or DENV cases in this study resulted from this cross-reactivity when the other flavivirus was, in fact, the causal agent. Despite this possibility, we found a much higher flavivirus IgM seroprevalence among this perinatal group than previously expected based on identified cases in recent years by the MOH. Dengvaxia DENV vaccine was not previously nor currently available in El Salvador at the time of participant sample collection, thus excluding the possibility of detecting vaccine derived antibodies vs. natural infection. Therefore, this study warrants future investigations of flavivirus in pregnancy, particularly in vulnerable regions of this highly endemic country.

In addition, ZIKV and DENV surveillance within the human population is not routinely conducted in El Salvador, therefore symptomatic cases presenting to health clinics are typically syndromically identified without laboratory confirmation. Likely due to overextension of the public health sector during the COVID-19 pandemic, surveillance and testing resources have been particularly strained in recent years. In 2021, only 2.7% of suspected DENV cases received confirmatory testing [24, 29]. Only 13.5% of 16,542 suspected cases of DENV received PCR or IgM testing in 2022 [26]. The number of suspected ZIKV infections that received clinical laboratory testing nationally is not published. Suspected and confirmed national cases of DENV and ZIKV can be found in Additional File 3. Due to the challenges in feasibility for sustained human surveillance along with the high rate of asymptomatic presentation of these flaviviruses, it is difficult to assess the true incidence of ZIKV and DENV in El Salvador. This study provides evidence that infections may be occurring more frequently than previously described and could present a continued threat to maternal-neonatal health in this country.

Our data warrants the need for education and laboratory screening for arboviral infections in the perinatal period, as infectious diseases and congenital malformations are the two leading causes of neonatal death in El Salvador, making this a major issue of public health importance [30]. Identification of pregnancies with high-risk of congenital transmission (early term ZIKV infection or late term DENV infection) may aid in early case detection of vertically infected neonates, to plan for proper continuum of neonatal care. Maternal screening could also benefit clinical management of maternal cases, particularly with DENV where maternal mortality risk is increased three-fold.

Almost all perinatal women in this study received at least one prenatal care visit in all three trimesters of pregnancy (96%), despite many women living in rural areas. This finding is in part, likely due to the recently enacted maternal-infant health care laws El Salvador. The 2022 “Ley Nacer con Cariño” (translated as Born with Care) has created novel infrastructure for perinatal health promotion and enhanced care for mothers and infants [25]. The government provides continuing education for OBGYN’s and healthcare professionals and quality inspections of labor and delivery units. The law also refocuses efforts towards enhanced prenatal care, promoting healthy fetal development, and comprehensive education for mothers and partners surrounding lactation and infant care.

As rural women often receive care by a health officer home visit, this health campaign presents a unique opportunity to incorporate further perinatal screenings for vertically transmissible infectious pathogens. Currently, HIV and syphilis are the only infectious pathogens mandatorily screened for in prenatal care [25], and screening for flaviviral infections may also be warranted in certain high-risk regions of the country. This point of intervention could also be utilized for expanded mosquito abatement and promotion of ITN-use adherence to aid in prevention among high-risk vulnerable women. Neighborhoods necessitating fumigation following a case of vector-borne disease could be prioritized for maternal flavivirus screening so that positive pregnancies can be monitored to aid in assuring positive outcomes for mother and baby. Spatially significant clustering of flaviviral IgM positivity occurred in Sonsonate and Cuisnahuat municipalities in Sonsonate department. These high-risk regions could be targeted for future arboviral infection studies and enhanced prenatal screening and education campaigns.

Though significant statistical associations were not found between seropositivity and adverse neonatal health outcomes, neonatal health status was assessed only at the time of birth. The authors hypothesize that due to a lack of hospital protocol to test for ZIKV infection combined with a limited neonatal assessment at birth, subclinical pathology cannot be excluded among these infants. APGAR and a basic physical check are the only two neonatal indicators assessed at this large referent hospital: neurologic function or head pathology beyond head circumference measurement are non-standard. Due to the high rate of ZIKV maternal positivity identified in this study during a MOH reported non-ZIKV outbreak year, further studies are warranted regarding arboviral infection in pregnancy in this region of El Salvador. Collectively this evidence suggests a possibility that congenital infections are ongoing, and maternal-fetal cases may be going undetected due to a lack of surveillance and the high rate of asymptomatic or non-specific hallmark symptomology.

Results of this study identified nearly one-quarter of women with evidence of a recent flavivirus infection at parturition, as evidenced by IgM + screening tests. As DENV and ZIKV IgM antibodies generally persist for only a few months following acute infection, results of this study suggest IgM + women in this study were potentially infected during their pregnancy. However, ZIKV and DENV IgM antibody decline and duration post-infection is not well characterized, particularly among adults, and few studies have attempted to quantify antibody kinetics for these infections [28, 31]. However, IgM following primary infection has been described more than one year following infection making temporality difficult to assess. In El Salvador, DENV is highly endemic with routine ZIKV outbreaks, thus this adult population is likely to have had prior arboviral infections. Therefore, a presumed shorter IgM duration (one representing infection within the third trimester of infection) is biologically possible in this population.

Future studies to clarify IgM antibody decline are critical in world regions where flavivirus is highly endemic, particularly in those countries where resource constraints limit confirmatory testing to primarily serologic methods. Further human surveillance studies are warranted among asymptomatic populations to clarify the true burden of these commonly asymptomatic arboviral infections, and to understand risk for vulnerable populations such as pregnant women.

This study screened banked maternal serum samples for flavivirus infection at parturition and analyzed health surveys to determine risk factors and health outcomes associated with infection. As 75–80% of ZIKV and DENV cases present asymptomatically, the true incidence of these infections is difficult to conclude. Further, there is a limited understanding of the clinical implications of asymptomatic flavivirus infection among pregnant women. This study provides a unique approach to better understanding both the incidence of asymptomatic infections and maternal-fetal outcomes among women with recent subclinical flavivirus infection during pregnancy.

This study has a few limitations worth noting. Specific pathogen serological results were not confirmed with plaque-reduction neutralization test nor by polymerase chain reaction, prompting statistical analysis to be performed among the collective flavivirus group due to inability to rule out potential antibody cross-reaction. Therefore unintended misclassification of DENV infections as ZIKV, based on IgM results alone, could dilute the ability to detect a significant relationship between congenital anomalies and maternal ZIKV infection. Secondly, due to the overall low number of municipalities and distribution of cases within our departments, the spatial statistic run to address case clustering should be taken as exploratory only. Future studies with higher recruitment across this region should be conducted to provide more rigorous autocorrelation and hotspot analyses. Additionally, incident infection timing cannot not be confirmed by DENV and ZIKV IgM antibody status; however, as these pathogens’ antibodies generally persist for a few weeks to months following acute infection [34, 35], it is possible that perinatal women in this study were infected during their pregnancy. Future investigations of antibody kinetics among susceptible adult populations are needed to assess infection temporality. Improved serologic assays that can be reliably employed in low-and-middle income countries, where these infections endemically circulate, are also critical to alleviate negative outcomes that stem from missed diagnoses of these infections.

## Conclusions

This report highlights the need for human arboviral surveillance among Salvadorans—the second largest immigrant group in the USA [36], particularly among pregnant women who are immunologically susceptible to negative health and neonatal birth outcomes when infection occurs [37]. Screening for neonatal infection or outcomes at childbirth outside the scope of APGAR and head circumference—neither are sensitive for ZIKV or DENV clinical presentation—are not routine in El Salvador. With up to 50% of ZIKV congenitally infected asymptomatic neonates developing neurodevelopmental delays by 2 years of age [38], it is imperative that maternal arboviral screening be implemented in this Central American country. Based on the results of this study, ZIKV infections may be continuing across the country at a higher rate than previously considered. The study’s geospatial analysis revealed statistically significant disease clusters, giving promise that targeted community interventions could reduce maternal arboviral transmission in this highly endemic region of El Salvador. Lastly, recently implemented laws to enhance maternal-child health, could provide a platform for maternal education and maternal flaviviral screening in high risk areas. Arboviral infections continue to be an important cause of maternal infection in El Salvador. Future studies are warranted to investigate asymptomatic maternal infections and corresponding neonatal health outcomes, pathogenic vertical transmission routes, and novel pregnancy-safe treatment options.

### Electronic supplementary material

Below is the link to the electronic supplementary material.

Supplementary Material 1: Arboviral IgM positive cases by municipality in Ahuachapan and Sonsonate, El Salvador.; Table of arbovirus cases defined by positive IgM result by municipality.

Supplementary Material 2: Cases of zika virus infection among pregnant women and children < 1 year 2015–2022, El Salvador.; Plot of probable or confirmed Zika virus and Dengue virus infection among infants less than one year of age and probable or confirmed Zika virus infection among pregnant women 2015 through 2022. Reported cases of infection were extracted from Salvadoran Ministry of Health Epidemiologic bulletins [25, 29, 39, 40].

Supplementary Material 3: Weekly epidemiologic bulletin synthesis of Zika and Dengue cases reported 2015–2022, El Salvador; Plot of suspected cases of Zika virus and Dengue virus infection, El Salvador 2015 through 2022. Reported cases were extracted from Salvadoran Ministry of Health Epidemiologic bulletins [25, 29, 39, 40].

## Data Availability

The datasets used and/or analyzed during the current study are available from the corresponding author on reasonable request.

## Abbreviations

ZIKV: Zika virus
DENV: dengue virus
MOH: Ministry of Health
ITN: Insecticide-treated bed net
NICU: neonatal intensive care unit
