# Supplementary material for: Perinatal dengue and Zika virus cross-sectional seroprevalence and maternal-fetal outcomes among El Salvadoran women presenting for labor-and-delivery
Source: Matern Health Neonatol Perinatol. 2024 Apr 2;10:7. doi: 10.1186/s40748-024-00177-5 (PMC10985905; doi:10.1186/s40748-024-00177-5)
Supplement: Supplementary file 1 — Supplementary Material 1: Arboviral IgM positive cases by municipality in Ahuachapan and Sonsonate, El Salvador.; Table of arbovirus cases defined by positive IgM result by municipality. [file 40748_2024_177_MOESM1_ESM.docx]

| **Department** | **Municipality** | **N participants** | **%(N) cases ZIKV IgM+ only** | **%(N) cases DENV IgM+ only** | **%(N) DENV and ZIKV IgM+** | **%(N) DENV and/or ZIKV IgM+** |
| --- | --- | --- | --- | --- | --- | --- |
| Ahuachapan | Guaymango | 1 | 0%(0) | 0%(0) | 0%(0) | 0.0%(0) |
|  | Jujutla | 8 | 12.5%(1) | 0%(0) | 0%(0) | 12.5% (1) |
|  | San Francisco Menendez | 20 | 25%(5) | 0%(0) | 0%(0) | 25.0% (5) |
|  | San Pedro Puxtla | 2 | 0%(0) | 0%(0) | 0%(0) | 0.0%(0) |
|  | Tacuba | 1 | 0%(0) | 0%(0) | 0%(0) | 0.0%(0) |
| Sonsonate | Acajutla | 21 | 23.8%(5) | 0%(0) | 9.5%(2) | 33.3 %(7) |
|  | Armenia | 6 | 0%(0) | 16.7%(1) | 0%(0) | 16.7%(1) |
|  | Caluco | 6 | 33.3%(2) | 0%(0) | 0%(0) | 33.3%(2) |
|  | Cuisnahuat | 8 | 25.0%(2) | 0%(0) | 12.5%(1) | 37.5%(3) |
|  | Izalco | 27 | 22.2%(6) | 3.7%(1) | 0%(0) | 25.9%(7) |
|  | Juayua | 6 | 16.7%(1) | 0%(0) | 0%(0) | 16.7%(1) |
|  | Nahuilingo | 2 | 50%(1) | 0%(0) | 0%(0) | 50.0%(1) |
|  | Nahuizalco | 25 | 24.0%(6) | 0%(0) | 0%(0) | 24.0%(6) |
|  | Salcoatitán | 4 | 25%(1) | 0%(0) | 0%(0) | 25.0%(1) |
|  | San Antonio del Monte | 8 | 25%(2) | 0%(0) | 0%(0) | 25.0%(2) |
|  | San Julian | 6 | 0%(0) | 0%(0) | 0%(0) | 0.0%(0) |
|  | Santa Catarina Masahuat | 2 | 0%(0) | 0%(0) | 0%(0) | 0.0%(0) |
|  | Santa Isabel Ishuatan | 8 | 25%(2) | 0%(0) | 0%(0) | 25.0%(2) |
|  | Santo Domingo de Guzman | 6 | 16.7%(1) | 0%(0) | 0%(0) | 16.7%(1) |
|  | Sonsonate | 24 | 16.7%(4) | 4.2%(1) | 8.3%(2) | 29.2%(7) |
|  | Sonzacate | 7 | 14.3%(1) | 0%(0) | 0%(0) | 14.3%(1) |
| Total: |  | 198 | 20.2%(N=40) | 1.5% (N=3) | 2.5% (N=5) | 24.2% (N=48) |

Additional file 1: Arboviral IgM positive cases by municipality in Ahuachapan and Sonsonate, El Salvador.

**% presented in this table are out of N participants from each municipality.*
