# Supplementary figures and images for: Perinatal dengue and Zika virus cross-sectional seroprevalence and maternal-fetal outcomes among El Salvadoran women presenting for labor-and-delivery
Source: Matern Health Neonatol Perinatol. 2024 Apr 2;10:7. doi: 10.1186/s40748-024-00177-5 (PMC10985905; doi:10.1186/s40748-024-00177-5)

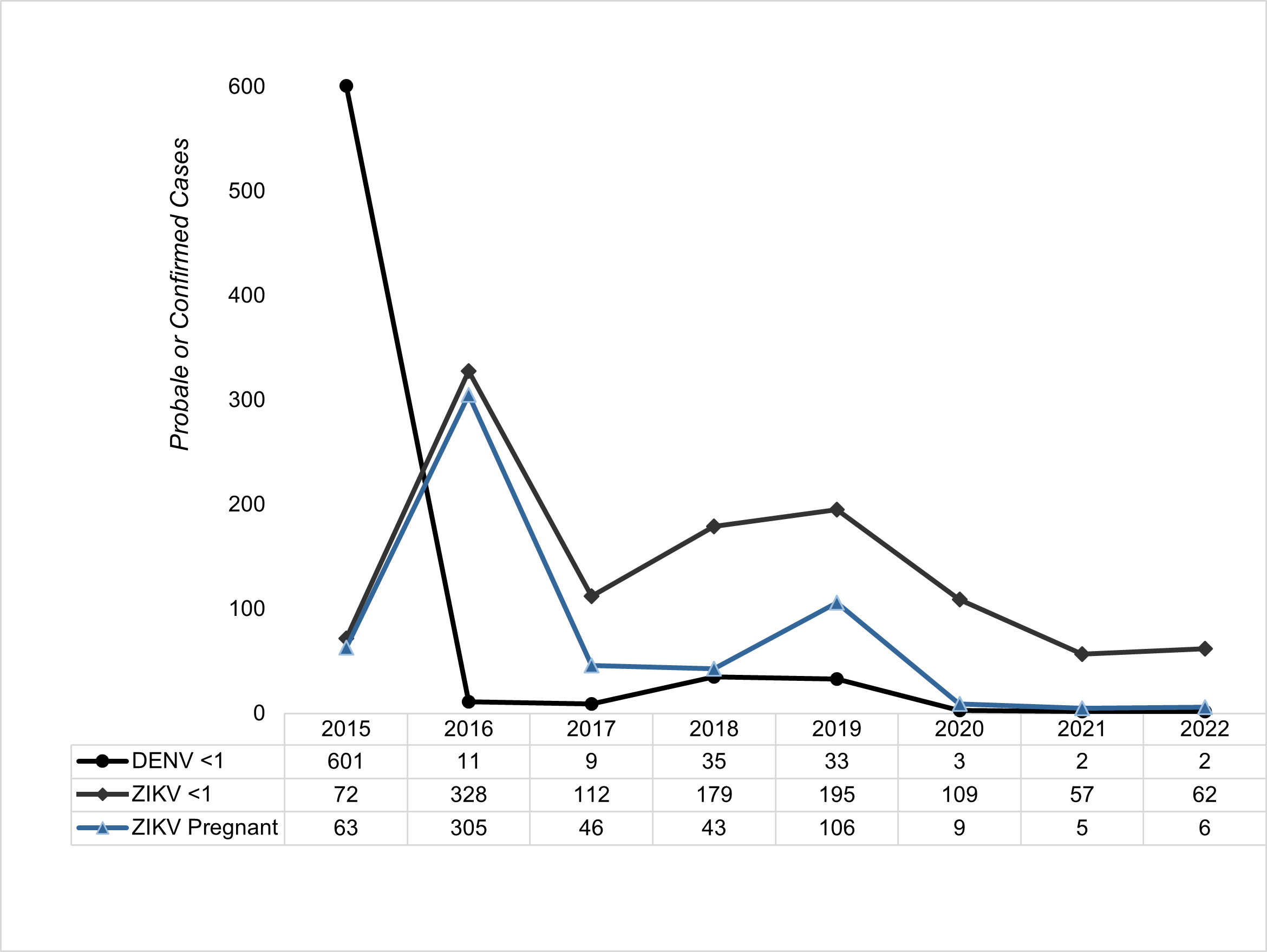

Supplement: Supplementary file 2 — Supplementary Material 2: Cases of zika virus infection among pregnant women and children < 1 year 2015–2022, El Salvador.; Plot of probable or confirmed Zika virus and Dengue virus infection among infants less than one year of age and probable or confirmed Zika virus infection among pregnant women 2015 through 2022. Reported cases of infection were extracted from Salvadoran Ministry of Health Epidemiologic bulletins [25, 29, 39, 40]. [file 40748_2024_177_MOESM2_ESM.tif]

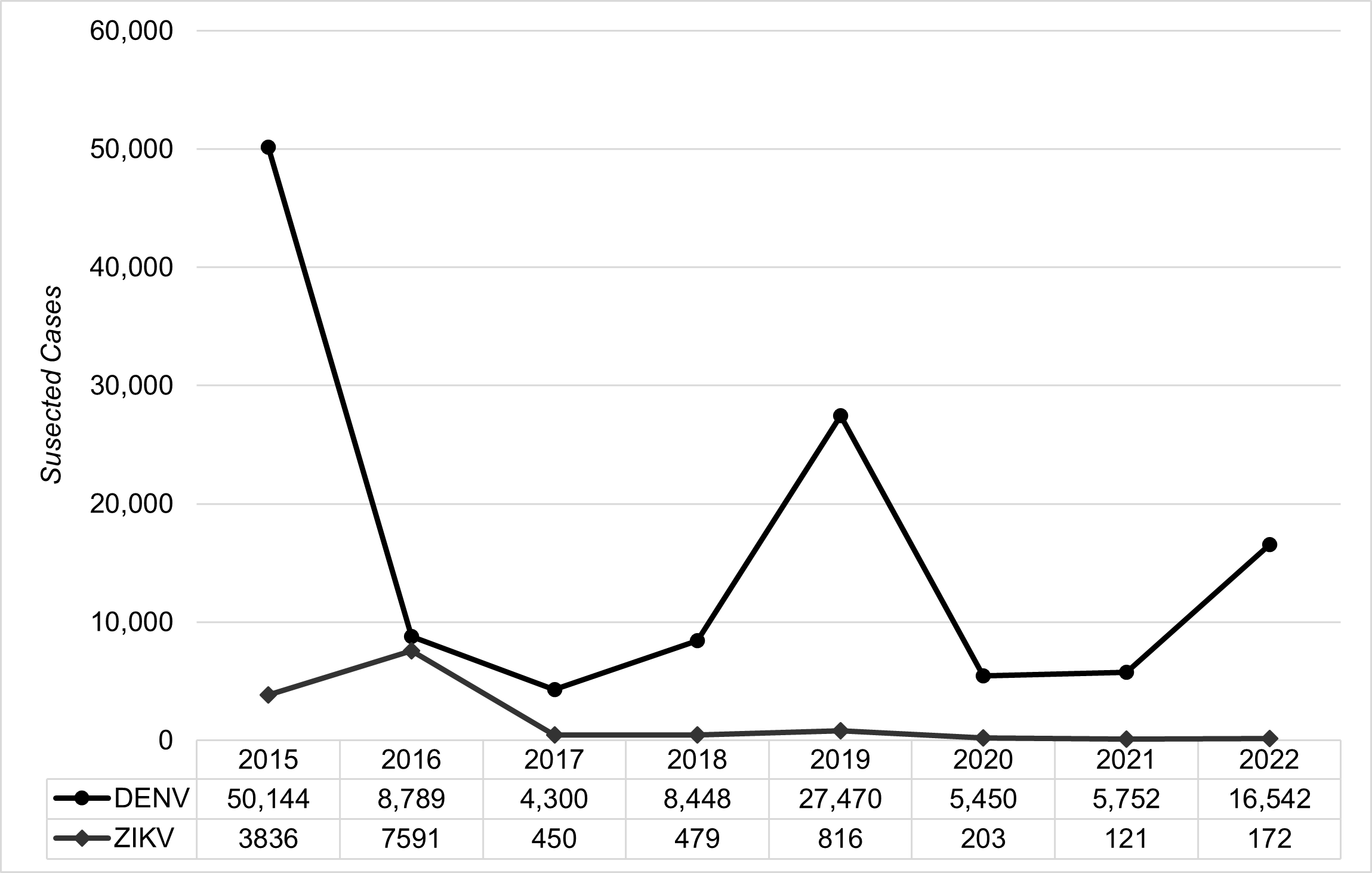

Supplement: Supplementary file 3 — Supplementary Material 3: Weekly epidemiologic bulletin synthesis of Zika and Dengue cases reported 2015–2022, El Salvador; Plot of suspected cases of Zika virus and Dengue virus infection, El Salvador 2015 through 2022. Reported cases were extracted from Salvadoran Ministry of Health Epidemiologic bulletins [25, 29, 39, 40]. [file 40748_2024_177_MOESM3_ESM.tif]
